# Relationships between Violence Experience, Resilience, and the Nursing Performance of Emergency Room Nurses in South Korea

**DOI:** 10.3390/ijerph19052617

**Published:** 2022-02-24

**Authors:** Sarang Kim, Minkyung Gu, Sohyune Sok

**Affiliations:** 1 Department of Nursing, Graduate School, Kyung Hee University, Seoul 02447, Korea; 11511@snubh.org; 2 Department of Nursing, College of Science and Technology, Daejin University, Pocheon-si 11159, Korea; g-minkyung@hanmail.net; 3 College of Nursing Science, Kyung Hee University, Seoul 02447, Korea

**Keywords:** workplace violence, resilience, nursing performance, emergency room nurse

## Abstract

In urgent situations where tensions and conflicts are amplified, emergency room nurses are vulnerable to violence and are exposed to dangerous situations because they are confronted by patients or caregivers. This study sought to examine the relationship between violence experience, resilience, and nursing performance among emergency room nurses in South Korea. A cross-sectional descriptive design was used. The study participants included 130 nurses working in the emergency room of a general hospital. Measures included the general characteristics list, the violence experience tool, the resilience tool, and the nursing performance tool. Data were collected from February to March 2021. In this study, among the forms of violence experienced by emergency room nurses, verbal violence was most prevalent. The violence experiences showed significant differences according to age, clinical experience, work experience in the emergency room, position, and job satisfaction. Resilience displayed significant differences according to marital status, clinical experience, position, average monthly salary, and job satisfaction. Nursing performance showed significant differences based on gender, age, marital status, clinical experience, work experience in the emergency room, position, average monthly salary, and job satisfaction. There was a positive correlation between resilience and nursing performance. This study suggests that emergency room nurses in Korea experienced more verbal violence than other types of violence. The violence experiences, resilience, and nursing performance showed significant differences according to the general and job-related characteristics of the study participants. Concrete strategies and interventions to reduce the frequency of experiences of verbal violence among emergency room nurses, increase their resilience, and improve the nursing performance of emergency room nurses are needed.

## 1. Introduction

In South Korea, the incidence of cardiovascular and cerebrovascular diseases and the incidence of emergency situations such as industrial accidents and traffic accidents are increasing [1]. Accordingly, the demand for emergency medical care has also increased [1,2]. The increase in demand for emergency medical services results in 24 h openness, frequent visits of critically ill patients, and delays in hospitalization, thus leading to friction between emergency room patients and medical personnel [3,4].

In South Korea’s emergency medical service system, patients and their caregivers can move freely and frequently within the hospital, so medical interference continues to be inflicted on medical personnel. Patients or caregivers who visit the emergency room are in a very unstable state, both mentally and physically, as a result of a sudden accident or illness. Therefore, it is urgently necessary to take measures to ensure safety in the emergency room [5,6,7].

Emergency room nurses must deal with emergency patients and provide nursing care by understanding the situation. In emergency situations, nurses need the knowledge and skills that are necessary for rapid and accurate assessment, intervention, and patient care, and must make urgent decisions to solve problems [8,9]. In urgent situations where tensions and conflicts are amplified, emergency room nurses are vulnerable to violence and are exposed to dangerous situations because they are confronted by patients or caregivers [2,10].

Meanwhile, it was found that the experience of violence from patients or their caregivers in the emergency room was closely related to the occurrence of medical accidents, and this had a negative effect on emergency room nurses [4]. Emergency room nurses experience various types of violence from patients or their caregivers, including verbal and physical violence, because they perform nursing tasks in close proximity to patients due to the nature of their job. Outbursts from patients or their caregivers in the emergency room are very frequent and cause the emergency room nurses to feel helpless and exhausted, such as lowering their motivation [4,6,10]. It is reported that emergency room nurses’ coping methods related to experiences of violence allow them to respond positively to stressful situations, as their resilience is higher [1]. Resilience refers to the overall ability of a person to respond flexibly to violent situations or urgent changes as a positive force that can be used as a springboard to overcome various types of adversity and changing situations [11]. Therefore, resilience can be defined as a potential force that effectively overcomes the various and frequent stressful situations experienced by emergency room nurses, and it is essential to raise individual resources and strengths [12,13]. In order to promote the healing and well-being of patients in the urgent situation of the emergency room environment, resilience is an essential requirement for emergency room nurses. This has a significant impact on nursing performance in terms of efficiently achieving the goals of the nursing organization [14].

Nursing performance refers to the degree to which work is efficiently performed in order to prevent medical errors based on professionalism and to achieve the common goal of the nursing organization when providing nursing care [15]. In order to enhance nursing performance, emergency room nurses must first be provided with an environment in which they can properly solve nursing problems based on emergency patient information [16,17]. Even if exposed to a dangerous environment, demonstrating excellent resilience can result in improved nursing performance [12,13]. Resilience is a positive factor that can effectively overcome adversity experienced by individuals and at the same time overcome the stress they face.

This study will make it possible to suggest specific ways to enhance the nursing performance of emergency room nurses who need clinical intuition in emergency situations where tension and conflict are amplified. It will be helpful in the application of evidence-based nursing necessary for emergency room environments.

The purpose of this study was to examine the relationship between violence experience from patients or their caregivers, resilience, and nursing performance among emergency room nurses in Korea. The aims of this study were (1) to identify the general characteristics and job-related characteristics of emergency room nurses; (2) to identify the type and frequency of violence from patients or their caregivers experienced by emergency room nurses; (3) to examine the degrees of violence experience from patients or their caregivers, resilience, and nursing performance of emergency room nurses; (4) to examine the differences in the degrees of violence experience from patients or their caregivers, resilience, and nursing performance according to the general characteristics of emergency room nurses; (5) to examine the correlations among the violence experience from patients or their caregivers, resilience, and nursing performance of emergency room nurses.

## 2. Material and Methods

### 2.1. Study Design and Participants

A cross-sectional descriptive design was used in this study. The study participants of this study were 130 nurses working in the emergency room of a general hospital. All emergency room nurses providing direct care to patients were included in the study. Emergency room nurses with less than 1 year of clinical experience were excluded from this study because it was judged that they lacked nursing knowledge about emergency patients and had insufficient experience in performing nursing. Head nurses who did not provide direct nursing care were also excluded from this study. To calculate the number of samples in this study, the number of samples calculated with a median effect size of 0.25, significance level of 0.05, and power of 0.8 by correlation analysis using the G-Power 3.1 program [18] was 123. Therefore, the number of samples in this study was satisfied. In the study, 130 (92.86%) were used for the final analysis, excluding 10 survey participations that were omitted through online specialized sites.

### 2.2. Measures

General and job-related characteristics of the study participants included of gender, age, marital status, clinical experience, work experience in emergency room, position, monthly income, and job satisfaction. This consisted of a total of 8 items.

For the violence experience tool, the emergency room nurse violence type and item tool modified by Kim and Lee [19] was used. This tool consists of a total of 16 items: verbal violence (4 items: ‘cursing’, ‘talking down’, ‘screaming’, ‘threatening’), physical threat (5 items: ‘taking a stance to hit’, ‘making a fierce look’, ‘taking a posture to throw things’, ‘kicking hospital items’, ‘wandering around with anger’), and physical violence (7 items: ‘throwing an object at me’, ‘striking or kicking me’, ‘pushing me’, ‘catching a part of my body’, ‘scratching’, ‘biting’, ‘spitting at me’). As a 5-point Likert scale, the score ranges from 4 to 20 points for verbal violence, 5 to 25 points for physical threats, and 7 to 35 points for physical violence. A higher score means more experience of violence from patients or their caregivers. In the study of Kim and Lee [19], the reliability of the tool was expressed in its Cronbach’s α = 0.87, and the reliability of this study was expressed in its Cronbach’s α = 0.91.

As the resilience tool, the tool developed by Park and Park [11] (Korean version of the Connor Davision Resilience Scale, KCDRISC) was used. This tool consists of items such as ‘I see the positive side of things when difficult things happen’ and ‘I do my best to solve problems’. This tool is a 4-point Likert scale with a total of 30 items. The score ranges from a minimum of 30 to a maximum of 120, with higher scores indicating higher resilience. The reliability of the tool at the time of development was expressed in its Cronbach’s α = 0.95, and the reliability of this study was expressed in its Cronbach’s α = 0.96.

The nursing performance tool was modified and used by Ko and Kim [20], and this tool was used in this study. This tool consists of a total of 17 items in 4 sub-domains, 7 items on work performance ability, 4 items on work performance attitude, 3 items on job level improvement, and 3 items on nursing process application. It consists of items such as ‘I have the knowledge and skills necessary to perform my duties’ and ‘I show interest and receptive attitude toward patients and caregivers’. On a 5-point Likert scale, the score ranges from a minimum of 17 to a maximum of 85, with a higher score indicating a higher degree of nursing performance. In the study of Ko and Kim [20], the reliability of the tool was expressed in its Cronbach’s α = 0.92, and the reliability of this study was expressed in its Cronbach’s α = 0.95.

### 2.3. Procedures

This study collected data from nurses working in the emergency room from February to March 2021, after approval by the Institutional Research Board of K University. For data collection, a questionnaire was constructed through an online specialized site, and a link for access to the questionnaire was posted on the online community along with the recruitment notice of research subjects. In this study, emergency room nurses were able to increase access to survey participation while reducing spatial and temporal constraints.

### 2.4. Statistical Analysis

The collected data were analyzed using SPSS Window 25.0 Program (IBM, Armonk, NY, USA) as follows according to the research purpose. The general characteristics and job-related characteristics of the study participants were analyzed in terms of frequency and percentage using descriptive statistics and frequency analysis. The frequency of each type of violence experience from patients or their caregivers was analyzed in terms of frequency and percentage using descriptive statistics and frequency analysis. The degrees of violence experience from patients or their caregivers, resilience, and nursing performance were analyzed using descriptive statistics as the mean and standard deviation. An independent *t*-test and one-way ANOVA were used to analyze the differences in violence experience from patients or their caregivers, resilience, and nursing performance according to the subjects’ general characteristics, and significant variables were analyzed by post-test using Scheffe. Correlations among the experiences of violence from patients or their caregivers, resilience, and nursing performance were analyzed with Pearson’s correlation coefficient.

### 2.5. Ethical Considerations

For ethical considerations, after obtaining approval from the Institutional Research Board of K University, the purpose and procedure of this study were explained to the study participants, and after obtaining consent for data collection, it was revealed that these data would not be used for any other purpose than the research purpose. Study participants were informed of their confidentiality, explaining that they can withdraw at any time during the study if they do not wish to.

## 3. Results

### 3.1. General and Job-Related Characteristics of the Study Participants

The general characteristics of the study participants and job-related characteristics of the study participants are shown in Table 1.

### 3.2. Types and Frequency of Violence Experience

In terms of verbal violence that occurred once a week, the most common experiences of the study participants were ‘cursing’ (72, 55.4%), ‘talking down’ (51, 39.2%), ‘screaming’ (59, 45.4%), and ‘threatening’ (57, 43.8%). In terms of physical threats that occurred once a month, the most common experiences of the study participants were ‘taking a stance to hit’ (55, 42.3%), ‘taking a posture to throw things’ (50, 38.5%), ‘kicking hospital items’ (63, 48.5%), and ‘wandering around with anger’ (47, 36.2%). Meanwhile, 40 study participants each (30.8%) experienced ‘making a fierce look’ once a month and twice a month, respectively. In terms of physical violence that occurred over the course of a year, 74 study participants (56.9%) had no experience of ‘throwing an object at me’ and 70 study participants (53.8%) had no experience of ‘striking or kicking me’. The most common experiences that the study participants had twice a year and once a year were ‘pushing me’ (41, 31.5%) and ‘catching a part of my body’ (43, 33.1%), respectively. However, the majority of the study participants did not experience ‘scratching’ (79, 60.8%), ‘biting’ (93, 71.5%), and ‘spitting at me’ (80, 61.5%) (Table 2).

### 3.3. Levels of Violence Experience, Resilience, and Nursing Performance of the Study Participants

When examining the study participants’ violence experience, resilience, and nursing performance, the total score for violence experience averaged 24.26, which was lower than the median value of 48 points. The average total score for resilience was 81.64, which was higher than the median value of 75 points. Nursing performance averaged 62.08 points, which was higher than the median value of 51 points (Table 3).

### 3.4. Differences in Violence Experience, Resilience, and Nursing Performance according to the General and Job-Related Characteristics of the Study Participants

In this study, the violence experiences according to the general characteristics of the study participant showed significant differences according to age (F = 5.633, *p* = 0.005), clinical experience (F = 2.580, *p* = 0.041), work experience in emergency room (F = 5.439, *p* = 0.005), position (t = 2.013, *p* = 0.046), and job satisfaction (F = 4.839, *p* = 0.009). In addition, it was found that the more dissatisfied with the working conditions a general nurse was, the more experiences of violence they reported.

In this study, resilience showed significant differences according to marital status (t = −2.605, *p* = 0.010), clinical experience (F = 4.772, *p* = 0.001), position (t = −2.932, *p* = 0.004), average monthly salary (F = 7.231, *p* < 0.001), and job satisfaction (F = 3.693, *p* = 0.028). In the case of married persons, resilience was found to be high, and those with more than 10 years of clinical experience showed relatively higher resilience. The difference by position showed that the resilience of the charge nurse was higher than that of the general nurse, and the higher the average monthly salary, the higher the resilience. In addition, the more satisfied with the job the nurse was, the higher the resilience.

Nursing performance in this study showed significant differences based on gender (t = −3.061, *p* = 0.003), age (F = 4.212, *p* = 0.017), marital status (t = −3.409, *p* = 0.001), clinical experience (F = 2.696, *p* = 0.034), work experience in the emergency room (F = 3.656, *p* = 0.029), position (t = −2.881, *p* = 0.005), average monthly salary (F = 6.091, *p* = 0.001), and job satisfaction (F = 5.864, *p* = 0.004). Nursing performance was higher in women than in men, and when they were under 30 years old and married. Those with more than 10 years of clinical experience and more than 5 years of work experience in emergency room showed higher nursing performance. Nursing performance was found to be higher for those with the charge nurse position and a higher average monthly salary. Finally, it was found that nursing performance was relatively higher when the nurses were satisfied with their job (Table 4).

### 3.5. Correlations among Violence Experience, Resilience, and Nursing Performance

The correlations among violence experience, resilience, and nursing performance did not show a significant correlation, but there was a positive correlation between resilience and nursing performance (γ = 0.610, *p* < 0.001). In other words, it was found that the higher the resilience of the study participants, the higher the nursing performance (Table 5).

## 4. Discussion

In this study, the frequency of verbal violence among the types of violence experienced by emergency room nurses in Korea was higher. In particular, more than half of the nurse surveyed experienced abusive language. The younger the nurse and the more limited their emergency room work experience, the more violence experience they reported. Resilience and nursing performance were found to be higher in those who were married, had more clinical experience, had a higher monthly salary, and were more satisfied with their job. There was a positive correlation between resilience and nursing performance.

Most of the emergency room nurses who participated in this study were unmarried in their early 30s, and most of them had less than 3 years of experience in the emergency room. Considering that more than half of the emergency room nurses have less than 3 years of experience, it can be seen that work stress related to emotional labor is high [21,22]. Therefore, continuous and effective management is needed to improve the nursing work environment, reduce the work stress of emergency room nurses, and provide psychological stability.

With regard to the types and frequency of violence experienced by emergency room nurses, verbal violence once a week was the most common. This is similar to the study results of Baek and Kang [23], Ju et al. [24], and Hwang and Han [22], who reported high levels of experience of verbal violence in the emergency room. In addition, studies by Kang and Park [25] and Park and Lee [26] showed consistent results with this study. They reported that verbal violence experienced by nurses working in special departments compared to other departments was higher than physical threats or violence directly exercised. As a result, there is an urgent need for safety measures to prevent and take action against verbal violence experienced by emergency room nurses [27].

In regard to the violence experienced by emergency room nurses, those who were younger, had less clinical and emergency room work experience, had a lower position (general nurses), and had lower the job satisfaction were more frequently exposed to violence experience. This result is contrary to the study by Ju et al. [24] reporting that the higher the education level, and the greater the clinical and emergency room work experience, the higher the frequency of exposure to violence experience. However, since the violence experienced by emergency room nurses can make anyone a direct victim, a systematic education program that can be considered as a correct response method necessary for recognizing violence and responding to dangerous situations of violence more quickly is required on a regular basis [28].

Next, the resilience of emergency room nurses who participated in this study showed significant differences according to marital status, clinical experience, position, average monthly salary, and job satisfaction. This is consistent with the results of the studies by Kim et al. [29] and Hwang and Han [22]. They reported that there are differences in clinical experience, average monthly salary, and job satisfaction depending on the size of the hospital organization, which can affect the resilience of nurses in relation to nurse treatment.

Based on these previous studies, specific working environment improvement measures to increase the resilience of emergency room nurses are required by specifically investigating the working conditions of emergency room nurses by medical institution. In addition, a support system within the hospital organization needs to be established in order to improve the treatment of emergency room nurses [30,31]. Nursing personnel need to be supplemented for screening patients with emergency and acute diseases, and special leave and allowances should be provided so that emergency room nurses can rest comfortably.

In this study, the nursing performance of emergency room nurses showed significant differences in most of the factors, such as gender, age, marital status, clinical experience and emergency room work experience, position, average monthly salary, and job satisfaction. These results support the study results of Hassankhani et al. [4] demonstrating that the working conditions of medical institutions and age-related organizational culture and the work skill and professionalism according to the work experience of nurses can greatly affect nursing performance. Recently, as attention has been focused on the quality of health care services, the importance of nursing performance is emphasized based on the expertise of nursing services.

Finally, in this study, it was found that there was a positive correlation between resilience and nursing performance, excluding experiences of violence by emergency room nurses, which was similar to the results of the studies on clinical nurses by Jang et al. [32] and Lee et al. [33]. In order to enhance the nursing performance of emergency room nurses, it is necessary to improve the environment for sufficient rest and insufficient manpower replenishment to increase the resilience of emergency room nurses and in the process of preparing and implementing emergency situations, and to accurately diagnose and supplement in detail the problems in nursing care according to the establishment of a systematic and efficient emergency medical system [7,34,35].

Identifying and properly addressing the violence experienced by emergency room nurses and increasing their resilience will be very effective in improving their nursing performance.

Based on the results of this study, there is an urgent need for specific interventions and programs to lower the violence experience of emergency room nurses who work in a stressful environment 24 h a day, increase resilience, and improve nursing performance. In addition, there is a need for measures from medical institutions and national support policies that will ensure the safety of nurses working in emergency rooms, particularly from verbal violence.

In order to achieve stable and efficient nursing manpower management and nursing performance in the emergency room in the future, further research is deemed necessary in order to analyze specific and detailed contents related to the experience of emergency room nurses’ turnover due to violence experiences and job stress. In particular, it is considered that a qualitative study relating to the causes, processes, and situations of burnout in the relationship between resilience due to violence experiences of emergency room nurses and nursing performance is required. Experimental research is also necessary in order to develop and verify a violence prevention nursing intervention program for emergency room nurses by expanding the relationship between experience of violence, resilience, and nursing performance of emergency room nurses.

Since the subjects of this study were conducted through emergency room nurses at a university hospital, there are limitations in explaining the degree of violence experience, resilience, and nursing performance of all emergency room nurses due to the limitation of the sample regarding emergency room nurses. Therefore, it is necessary to conduct an expanded study in consideration of the sample of study subjects in the future.

## 5. Conclusions

Based on the results of this study, in order to achieve high-quality nursing performance for nurses working in emergency rooms, measures from medical institutions and national support policies are needed to ensure safety from verbal abuse. In addition, concrete measures and intervention programs to increase the resilience of emergency room nurses who work under pressure 24 h a day are urgently needed. It will be possible to seek an intervention plan that can reduce the frequency of experiences of verbal violence of emergency room nurses, increase resilience, and improve nursing performance, which could ultimately improve the safety and quality of nursing for emergency room nurses.

## Figures and Tables

**Table 1:** General and job-related characteristics of the study participants (*n* = 130).

Characteristics	*n*	%
Gender		
Male	13	10.0
Female	117	90.0
Age		
<30	40	30.8
30–34	59	45.4
≥35	31	23.8
Marital status		
Married	30	23.1
Single	100	76.9
Clinical experience (year)		
<3	26	20.0
3–5	48	36.9
6–7	25	19.2
8–10	14	10.8
>10	17	13.1
Work experience in emergency room (year)		
<3	89	68.5
3–5	25	19.2
>5	16	12.3
Position		
General nurse	115	88.5
Charge nurse	15	11.5
Monthly income (10,000 won)		
<250	15	11.5
250–300	44	33.8
301–350	60	46.2
>350	11	8.5
Job satisfaction		
Bad	40	30.8
Moderate	63	48.5
Good	27	20.7

**Table 2:** Types and frequency and types of violence experience.

Variables	Types	None	1 Time	2 Times	3 Times	4 or More Times
		*n* (%)	*n* (%)	*n* (%)	*n* (%)	*n* (%)
	Cursing	7 (5.4)	72 (55.4) ^1^	34 (26.2)	12 (9.2)	5 (3.8)
Verbal	Talking down	2 (1.5)	51 (39.2) ^1^	35 (26.9)	20 (15.4)	22 (16.9)
violence	Screaming	3 (2.3)	59 (45.4) ^1^	38 (29.2)	19 (14.6)	11 (8.5)
(per week)	Threatening	38 (29.2)	57 (43.8) ^1^	17 (13.1)	16 (12.3)	2 (1.5)
	Taking a stance to hit	42 (32.3)	55 (42.3) ^1^	20 (15.4)	12 (9.2)	1 (0.8)
Physical	Making a fierce look	14 (10.8)	40 (30.8) ^1^	40 (30.8) ^1^	25 (19.2)	11 (8.5)
threat	Taking a posture to throw things	34 (26.2)	50 (38.5) ^1^	32 (24.6)	13 (10.0)	1 (0.8)
(per month)	Kicking hospital items	25 (19.2)	63 (48.5) ^1^	24 (18.5)	14 (10.8)	4 (3.1)
	Wandering around with anger	9 (6.9)	47 (36.2) ^1^	46 (35.4)	17 (13.1)	11 (8.5)
	Throwing an object at me	74 (56.9) ^1^	30 (23.1)	23 (17.7)	3 (2.3)	0 (0.0)
Physical	Striking or kicking me	70 (53.8) ^1^	35 (26.9)	23 (17.7)	2 (1.5)	0 (0.0)
violence	Pushing me	37 (28.5)	35 (26.9)	41 (31.5) ^1^	13 (10.0)	4 (3.1)
(per year)	Catching a part of my body	31 (23.8)	43 (33.1) ^1^	37 (28.5)	14 (10.8)	5 (3.8)
	Scratching	79 (60.8) ^1^	27 (20.8)	18 (13.8)	5 (3.8)	1 (0.8)
	Biting	93 (71.5) ^1^	23 (17.7)	11 (8.5)	2 (1.5)	1 (0.8)
	Spitting at me	80 (61.5) ^1^	26 (20.0)	17 (13.1)	7 (5.4)	0 (0.0)

^1^ The greatest frequency and percentage.

**Table 3:** Levels of violence experience, resilience, and nursing performance of the study participants.

Variables	Range Point (Median)	Total Score Average Mean ± SD
Violence experience	16–80 (48)	24.26 ± 12.79
Verbal violence	4–20 (12)	8.15 ± 3.98
Physical threat	5–25 (15)	8.98 ± 4.93
Physical violence	7–35 (21)	7.13 ± 5.85
Resilience	30–120 (75)	81.64 ± 15.26
Nursing performance	17–85 (51)	62.08 ± 10.26

**Table 4:** Differences on violence experience, resilience, and nursing performance according to general and job-related characteristics of the study participants.

Characteristics	Violence Experience		Resilience		Nursing Performance	
	Mean ± SD	t or F (*p*) Scheffe	Mean ± SD	t or F (*p*) Scheffe	Mean ± SD	t or F (*p*) Scheffe
Gender		0.028 (0.978)		−1.586 (0.115)		−3.061 (0.003 *)
Male	19.46 (6.94)		75.30 (14.10)		54.06 (11.22)	
Female	19.40 (10.55)		82.20 (15.30)		62.90 (9.86)	
Age		5.633 (0.005 *) a,b > c		2.804 (0.064)		4.212 (0.017 *) a > b
<30	21.33 (10.22) a		86.10 (12.90) a		65.45 (9.52) a	
30–34	20.83 (10.19) b		78.90 (15.00) b		59.50 (9.69) b	
≥35	14.23 (8.73) c		81.00 (17.40)		62.73 (11.22) c	
Marital status		0.126 (0.900)		−2.605 (0.010 *)		−3.409 (0.001 *)
Yes	19.47 (10.33)		87.90 (16.20)		67.49 (9.18)	
No	19.20 (10.03)		79.80 (14.40)		60.52 (10.03)	
Clinical experience (years)		2.580 (0.041 *) a,b,c > d,e		4.772 (0.001 *) b,e > d		2.696 (0.034 *) d < e
<3	21.00 (13.42) a		81.90 (15.90) a		61.20 (10.88) a	
3–5	20.83 (9.26) b		83.10 (13.20) b		62.39 (9.86) b	
6–7	21.08 (8.12) c		79.50 (15.90) c		60.69 (9.86) c	
8–10	13.07 (10.62) d		68.70 (11.70) d		57.29 (9.52) d	
>10	15.71 (7.43) e		90.60 (14.40) e		68.51 (9.35) e	
Work experience in emergency room (years)		5.439 (0.005 *) a > b,c		1.620 (0.202)		3.656 (0.029 *) a < c
<3	21.34 (10.70) a		80.10 (15.00)		60.52 (10.37) a	
3–5	15.68 (7.68) b		84.30 (15.90)		64.26 (7.99) b	
>5	14.50 (7.91) c		86.10 (15.60)		67.15 (10.71) c	
Position		2.013 (0.046 *)		−2.932 (0.004 *)		−2.881 (0.005 *)
General nurse	20.05 (10.37)		80.40 (15.00)		61.20 (9.86)	
Charge nurse	14.47 (7.60)		92.10 (14.10)		69.02 (10.37)	
Monthly income (10,000 won)		1.335 (0.266)		7.231 (<0.001 *) a,b,c < d		6.091 (0.001 *) a,b,c < d
<250	23.00 (10.07)		80.10 (15.30) a		59.84 (11.39) a	
250–300	20.20 (10.33)		82.20 (13.50) b		62.90 (9.86) b	
301–350	18.62 (10.55)		78.30 (15.00) c		60.01 (9.52) c	
>350	15.64 (7.07)		99.90 (10.80) d		73.10 (6.97) d	
Job Satisfaction		4.839 (0.009 *) a > c		3.693 (0.028 *) a < c		5.864 (0.004 *) a < c
Bad	23.13 (8.64) a		77.10 (11.10) a		58.48 (10.37) a	
Moderate	18.62 (10.98) b		82.20 (15.00) b		62.39 (9.18) b	
Good	15.74 (9.07) c		87.00 (19.20) c		66.81 (10.71) c	

* *p* < 0.05. a, b, c, d show the post hoc test using Scheffe.

**Table 5:** Correlations among violence experience, resilience, and nursing performance. (*N* = 130).

Variables	Violence Experience	Resilience	Nursing Performance
	γ (*p*)		
Violence experience	1		
Resilience	0.152 (0.084)	1	
Nursing performance	0.107 (0.226)	0.610 (<0.001 *)	1

* *p* < 0.05.

## Data Availability

No new data were created or analyzed in this study. Data sharing is not applicable to this article.
